# Hospitals and Their Regulations

**Published:** 1836

**Authors:** 


### Art. IV. Hospitals and their regulations.

It would seem from a notice of the London Hospital, by Dr. Johnson, that clinical lectures are delivered in them in the same manner that they are in our own.

"Students paying at the rate of 25 guineas each to the principal surgeon of a hospital for their instruction, are permitted to follow the heels of the latter through the different wards—from 100 to 200 at least do this at a large hospital annually. It is plain, that so great a number cannot, as things are now conducted derive much benefit from their *soi-disant* instructors in what is called hospital practice. For how can even a fourth part of the number of students witness the conduct, or hear the opinions of the surgeon, at the patient's bedside? Ten or twelve get close to him, surround the bed, and exclude the rest from perceiving what is going on. The celerity too, with which the business of visiting the sick in hospitals is performed, adds to the pupil's difficulties. Now, the only mode in which the surgeon could repair this error, in receiving more than he can give a fair equivalent for, in the ward-hunting business, would be by delivering, daily, clinical lectures in the theatre of the school, where there would be accommodation for all—an hour, at least, daily, should the surgeon lecture."

We noticed the state of things here mentioned in the hospital in our own city, but we hoped that a different arrangement prevailed elsewhere. It seems, however, that it is as bad in London. The "ten or twelve" who surround the surgeon are not always the most studious. They are often pushed forward by curiosity rather than a spirit of inquiry, and hence they cannot detail what they have heard and seen fifteen minutes after they have left the wards. Even if they should remember all, it is forced upon them in such quantities that they cannot digest it. A single case is sufficient for the study of the most able beginner, for an entire day, but here he is not only furnished with twenty different cases, but he is hurried from the ward into the lecture room of the college, to hear lectures for four hours on Anatomy, Chemistry, Materia Medica, and perhaps Midwifery. Of what benefit is such clinical instruction to students? The truth is, from the way in which our hospitals are managed, few students visit them at all after the novelty of the thing wears away. Why is this state of things allowed to exist? Certainly hospitals might be so regulated as to be useful to students, as practical schools of clinical instruction. W. W.

